# Characterization of a Broad-Host-Range Lytic Phage SHWT1 Against Multidrug-Resistant *Salmonella* and Evaluation of Its Therapeutic Efficacy *in vitro* and *in vivo*

**DOI:** 10.3389/fvets.2021.683853

**Published:** 2021-06-10

**Authors:** Chenglin Tao, Zhengfei Yi, Yaodong Zhang, Yao Wang, Hong Zhu, Dossêh Jean Apôtre Afayibo, Tao Li, Mingxing Tian, Jingjing Qi, Chan Ding, Song Gao, Shaohui Wang, Shengqing Yu

**Affiliations:** ^1^Department of Animal Public Health, Shanghai Veterinary Research Institute, Chinese Academy of Agricultural Sciences, Shanghai, China; ^2^College of Veterinary Medicine, Yangzhou University, Yangzhou, China; ^3^Jiangsu Co-innovation Center for Prevention and Control of Important Animal Infectious Diseases and Zoonosis, Yangzhou, China

**Keywords:** *Salmonella*, bacteriophage, characteristics, biofilm, genome, multidrug-resistant

## Abstract

Inappropriate use of antibiotics has accelerated to the emergence of multidrug-resistant bacteria, becoming a major health threat. Moreover, bacterial biofilms contribute to antibiotic resistance and prolonged infections. Bacteriophage (phage) therapy may provide an alternative strategy for controlling multidrug-resistant bacterial infections. In this study, a broad-host-range phage, SHWT1, with lytic activity against multidrug-resistant *Salmonella* was isolated, characterized and evaluated for the therapeutic efficacy *in vitro* and *in vivo*. Phage SHWT1 exhibited specific lytic activity against the prevalent *Salmonella* serovars, such as *Salmonella* Pullorum, *Salmonella* Gallinarum, *Salmonella* Enteritidis, and *Salmonella* Typhimurium. Morphological analysis showed that phage SHWT1 was a member of the family *Siphoviridae* and the order *Caudovirales*. Phage SHWT1 had a latent period of 5 min and burst size of ~150 plaque-forming units (PFUs)/cell. The phage was stable from pH 3-12 and 4–65°C. Phage SHWT1 also showed capacity to lyse *Salmonella* planktonic cells and inhibit the biofilm formation at optimal multiplicity of infection (MOI) of 0.001, 0.01, 0.1, and 100, respectively. In addition, phage SHWT1 was able to lyse intracellular *Salmonella* within macrophages. Genome sequencing and phylogenetic analyses revealed that SHWT1 was a lytic phage without toxin genes, virulence genes, antibiotic resistance genes, or significant genomic rearrangements. We found that phage SHWT1 could successfully protect mice against *S*. *enteritidis* and *S*. *typhimurium* infection. Elucidation of the characteristics and genome sequence of phage SHWT1 demonstrates that this phage is a potential therapeutic agent against the salmonellosis caused by multidrug-resistant *Salmonella*.

## Introduction

*Salmonella* is a Gram-negative bacterium that is an important pathogen of humans and animals. *Salmonella* can cause numerous diseases, ranging from a self-limiting gastroenteritis to typhoid-like disease to systemic infections, known as salmonellosis. In addition, *Salmonella* is a leading etiological agent associated with outbreaks of food-borne illness worldwide, commonly resulting in global economic losses (1–3). Animals, including poultry are a main reservoir for *Salmonella*, such as the prevalent *Salmonella* serovars *Salmonella* Pullorum (*S*. Pullorum), *Salmonella* Gallinarum (*S*. Gallinarum), *Salmonella* Enteritidis (*S*. Enteritidis) and *Salmonella* Typhimurium (*S*. Typhimurium) (4–10). Some of these serovars can lead to serious zoonotic diseases via contaminated foods such as poultry and eggs.

Traditionally, antibiotics are used to prevent and treat *Salmonella* infections. However, the preventive antibiotics administration can accelerate the emergence of multidrug-resistant bacteria, including *Salmonella* (4, 11, 12). In addition, *Salmonella* can form biofilms on biotic or abiotic surfaces, which contribute to antibiotic resistance and prolonged infections. The increasing prevalence of multidrug-resistant bacteria means antibiotic treatments are becoming ineffective (13, 14). Consequently, there is an urgent need to develop alternative approaches to prevent and control salmonellosis caused by multidrug-resistant *Salmonella*.

Bacteriophages (phages) are specific viruses found in diverse environments that target and kill bacteria, and since their discovery, phages have been used to treat bacterial infections (15). Due to their wide distribution, it is easier to obtain and isolate phages than to discover new antibiotics. Moreover, phages are highly specific and only eliminate target bacteria without destroying the normal microbial flora. Thus, phages have received renewed attention as an alternative therapeutic option against infectious bacterial diseases due to the rise in antibiotic resistance (16–18). Phage therapy has been effectively applied to control bacterial infections of humans, animals, and plants as well as being used as a biological control for food-borne pathogens (19–23). Various phages with specific activity against *Salmonella* have been isolated and characterized (24–27). Moreover, commercial phage products, such as Salmonelex and SalmoFresh, have been approved as food-processing aids to control *Salmonella* in food products (28, 29).

However, there are some challenges with phage therapy, including the low efficacy, narrow host range, and emergence of phage-resistant bacteria (30, 31). Therefore, it is necessary to continuously search and characterize new phages with broad host range and high lytic capacity for further applications. In this study, a broad-host-spectrum phage, SHWT1, with lytic activity against multidrug-resistant *Salmonella* was isolated. Genome sequence analysis and characterization of phage SHWT1 was conducted. Furthermore, the efficiency of phage SHWT1 against *Salmonella* planktonic cells, biofilm formation, intracellular *Salmonella* within macrophages *in vitro* and *Salmonella* infection *in vivo* were investigated to explore potential use of the phage for biocontrol of salmonellosis.

## Materials and Methods

### Bacterial Strains and Growth Conditions

The bacterial strains, containing *Salmonella, Escherichia coli*, and *Staphylococcus aureus* were isolated from chicken farms in eastern China through selective medium culture and PCR confirmation in this study and our previous studies (32, 33). These bacteria were used for phage isolation and host range determination. *Salmonella* and *Escherichia coli* were routinely grown at 37°C in Luria-Bertani (LB) broth (pH 7.2-7.4) with shaking or on LB agar. *Staphylococcus aureus* was culture in Tryptic Soy broth (TSB) or on plates containing Tryptic Soy agar (TSA).

### Antibiotic Susceptibility

Susceptibility of the bacterial isolates to 18 different antibiotics was determined by the standard Kirby-Bauer test, with data analyzed according to guidelines of the Clinical and Laboratory Standards Institute (CLSI) (34).

### Isolation and Purification of Bacteriophages

Wastewater samples were collected from chicken farms in eastern China. Isolation and purification of phages were performed as described previously (35). Briefly, particulate matter in the wastewater samples was removed by centrifugation at 1,000 g. The supernatant was mixed with an *S*. Pullorum SP01 culture and incubated for 12 h at 37°C to enrich for phages. The enriched culture was centrifuged at 7,000 g for 10 min at 4°C, and supernatant was filtered using a 0.22-μm filter membrane (Millipore, USA) to remove bacteria. The filtrate was inoculated with the indicator *S*. Pullorum SP01 and seeded on soft agar plates according to the double-agar overlay method. Purified phages were obtained by performing a plaque assay six times. Phages were subsequently concentrated in SM buffer (100 mM NaCl, 8 mM MgSO_4_, 50 mM Tris-HCl) and stored at 4°C until further analyses.

### Determination of Phage Host Range

The bacterial isolates were tested for their susceptibility to phages using spotting methods as previously described (36). Then, the host range of phage were confirmed by double-agar overlay methods to avoid the so-called lysis from without (37).

### Phage Morphology

Phage morphology was determined by transmission electron microscopy (TEM). Briefly, purified phage was dropped on acopper grid and negatively stained with 2% phosphotungstic acid. The grids were then observed under a FEI T12 transmission electron microscope (FEI, Ltd, Hillsboro, OR, USA).

### Optimal Multiplicity of Infection (MOI) of Phage

The optimal MOI of phage was determined as previously described (38) with some modifications. The bacteria were grown to exponential phase and harvested. The pellets were suspended and adjust to OD600 nm as 0.2 (~1 ×10^8^ clone-forming units (CFUs)/mL) with an Eppendorf BioSpectrometerBasic Spectrophotometer (Eppendorf AG, Germany). The fresh culture of bacteria were mixed with equal volumes of diluted phage (1 ×10^3^-1 ×10^11^ plaque-forming units (PFUs)/mL) at the MOI as 0.00001, 0.0001, 0.001, 0.01, 0.1, 1, 10, and 100. Mixtures were cultured at 37°C for 3 h, then phage titer determination was performed using the double-layer plate method. Experiments were performed independently three times.

### Adsorption Rate and One-Step Growth Curve Assay

The adsorption ability of phage was determined as previously described (39) with some modifications. Briefly, the phage was mixed with host strain *S*. Pullorum SP01 (1 ×10^8^ CFUs/mL) at MOI of 0.1, and incubated at 37°C. The samples were taken at 1, 3, 5, and 10 min, and centrifuged at 10,000 g (4°C) to remove the absorbed phages. The titers of unabsorbed phages in the supernatant were determined after serial dilution. Finally, the percentages of phage adsorption at different time points were calculated. The adsorption rate constant (*k* value) of phage were calculated as previously described (40). Experiments were performed independently three times.

A one-step growth curve assay was performed as previously described with some modifications (38). The phage was mixed with exponential phase *S*. Pullorum SP01 (1 ×10^8^ CFUs/mL) at the MOI of 0.1, and was incubated at 37°C for 5 min to allow maximum adsorption of phage. Subsequently, the mixture was centrifuged at 10,000 g (4°C) for 10 min to remove unabsorbed phage. Then, the supernatant was discarded, and the pellets were washed and suspended with LB broth. These samples were incubated at 37°C and taken at indicated time points. The phage titers were measured using the double-layer agar method. Experiments were performed independently three times.

### Thermal and pH Stability

For thermal stability testing, the phage suspension was incubated at 4, 37, 40, 45, 50, 55, 60, 65, 70, 75, 80, and 85°C, respectively. Samples were taken after 20, 40, and 60 min and the phage titers were determined. In addition, the phage suspension was stored at room temperature (25°C) and phage titers were monitored for 4 weeks.

To investigate pH stability, phages were incubated at 37°C for 1 h in LB medium at pH ranging from 3 to 12 (adjusted with HCl or NaOH for acidic or alkaline, respectively). Phage titer from each pH were determined by the double-layered agar plate method as previously described (41). Experiments were repeated three times.

### Bacterial Challenge Test With Planktonic Cells

The bacterial challenge test was performed according to the protocol previously reported with minor changes (42). Representative *Salmonella* strains were grown to an OD600 nm of 0.2, and were infected with phage SHWT1 at the optimal MOI. Bacterial growth was determined by monitoring the optical density at 2 h intervals. Meanwhile, the number of bacterial cells were also determined by serial dilutions and plating.

### Determination of Bacterial Biofilm Susceptibility to Phage SHWT1

The effects of phage SHWT1 on biofilm formation by *Salmonella* strains were tested referring to previous studies (42, 43). Bacterial cultures with or without phage SHWT1 were dispensed in 96-well plates and cultured without shaking at 37°C for 24 or 48 h. Planktonic bacterial cells were removed and biofilms were stained with 200 μL crystal violet (0.1%, W/V) for 20 min. After rinsing and air-drying, the biofilms were solubilized, and the absorbance was measured at 595 nm. Given that most bacteria in clinical infections reside in biofilms, we tested the roles of phage SHWT1 on the number of bacteria within the biofilm as described previously (44). After 24 h incubation, the wells were washed twice with PBS to remove the planktonic bacterial cells. Then, the biofilms at the wells were disrupted, following by suspended and mixed in PBS. These biofilm suspensions were serial diluted and the bacteria were enumerated by plating. Experiments were performed three times.

In addition, the capacity to disrupt the bacterial biofilm was determined as previously study (42, 43). The *Salmonella* biofilms were developed for 24 h at 37°C as mentioned above. The wells were then washed twice and treated with phage SHWT1 or SM buffer control. After incubation, the supernatants were removed and wells were washed twice. Determination of biofilms and enumeration of bacteria within the biofilm were performed as mentioned above.

### Evaluation of the Intracellular Lytic Activity of Phage SHWT1

The lytic activity of phage SHWT1 against intracellular *Salmonella* were evaluated as described previously (45) with some modifications. In brief, the avian macrophage HD11 monolayers were washed with Dulbecco's modified Eagle's medium (DMEM) without fetal bovine serum (FBS), followed by infection with *Salmonella* strains at a multiplicity of infection (MOI) of 10 for 1 h at 37°C to allow maximum uptake. The infected cells were treated with DMEM containing gentamicin (100 μg/mL) for 1 h to kill extracellular bacteria. Then, the phage SHWT1 (10^8^ PFUs/well) was added into the cells for another 12 h treatment. The cells were washed and lysed with 0.5% Triton X-100, and the intracellular bacteria were enumerated by plating on LB plates.

### Genome Sequencing and Analysis

Phage DNA was extracted using the Phage Genome DNA Quick Extraction Kit (Zhuangmeng International Biology Gene Technology co., Ltd), and whole-genome sequencing was performed at the Shanghai Personal Biotechnology Co., Ltd on the Illumina NovaSeq PE150 platform with ~22,978-fold coverage. High-quality paired-end reads were assembled using A5-MiSeq v20160825 (https://arxiv.org/abs/1401.5130) and SPAdes v3.12.0 (http://cab.spbu.ru/files/release3.12.0/manual.html), and the genome sequence was proofread using software MUMmer v3.1 (http://mummer.sourceforge.net/) (46) and Pilon v1.18 (https://github.com/broadinstitute/pilon) (47). Potential open reading frames (ORFs) were predicted using GeneMarkS (48). Genome annotation was analyzed using RAST (https://rast.nmpdr.org/), HHpre (https://toolkit.tuebingen.mpg.de/#/tools/hhpred), BLAST and Conserved Domain Identify of NCBI. The Virulence Factor Database (http://www.mgc.ac.cn/VFs/main.htm) and Comprehensive Antibiotic Resistance Database (https://card.mcmaster.ca/) were queried to retrieve the toxic genes, virulence genes and antibiotic resistance genes in the phage genome. tRNAscan-SE search program (https://lowelab.ucsc.edu/tRNAscan-SE/) was used to identify putative tRNAs (49). A circular representation of the genome of phage SHWT1 was generated using BRIG software. Comparisons and phylogenetic analysis of the genome of phage SHWT1 with other phages were conducted with NCBI BLASTN algorithm (http://blast.ncbi.nlm.nih.gov). The complete genome sequence of phage SHWT1 has been deposited in the GenBank database under accession number MT740291.1.

### Ethics Statement

The animal experiments were conducted in strict accordance with the guidelines of the Humane Treatment of Laboratory Animals and were approved by the Animal Care and Use Committee at the Shanghai Veterinary Research Institute (permit No: SHVRI- SV-20201014-04).

### Efficacy of Phage Therapy for *Salmonella* Infection Using Mouse Model

To evaluate the therapeutic efficacy of phage SHWT1 *in vivo*, 6 week-old specific-pathogen-free BALB/c mice were infected with *S*. Enteritidis SE12 or *S*. Typhimurium SAT52 by oral administration. At the 6 or 12 h post-infection, mice were treated with a single dose (1.6 ×10^10^ PFUs) of phage SHWT1 by oral administration. As control groups, mice were challenged with PBS and received only the phage. The mortality of mice was monitored daily for 21 days.

### Statistical Analyses

Statistical analyses were conducted using the GraphPad Software package. Multivariate comparisons were analyzed by using one-way and two-way analysis of variance (ANOVA). Values were considered significant when *P* < 0.05.

## Results

### Determination of the Antibiotic Sensitivity Profile of Bacterial Isolates

Over the study period, various bacteria were isolated and identified by PCR, including *Salmonella, Escherichia coli*, and *Staphylococcus aureus*. Determination of antibiotic sensitivity revealed the isolates had different antibiotic resistance profiles. In general, the isolates were resistant to 6–18 antibiotics (Supplementary Table 1), which indicated that the isolates were multidrug-resistant.

### Isolation, Purification, and Host Range Determination of Phages

**Figure 1:**
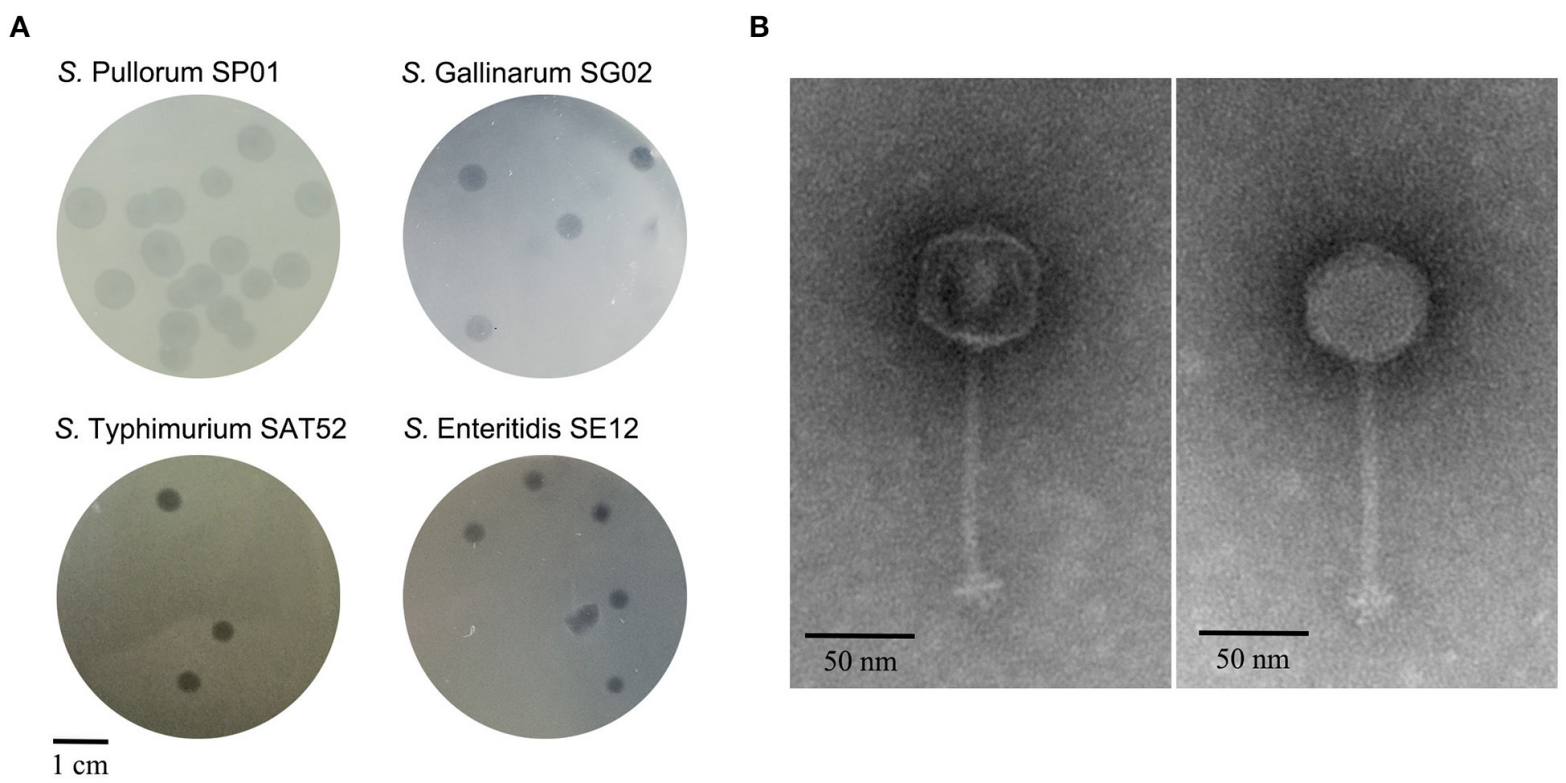
Morphology of phage SHWT1. **(A)** Plaques morphologies of phage SHWT1. Phage SHWT1 formed clear plaques with well-defined boundaries on bacterial lawns of *S*. Pullorum, *S*. Gallinarum, *S*. Enteritidis, and *S*. Typhimurium. Scale bar, 1 cm. **(B)** Transmission electron micrographs of phage SHWT1. Scale bar, 50 nm.

Sixty-eight lytic phages infecting *S*. Pullorum were isolated and purified from the wastewater samples collected from chicken farms in eastern China. These phage isolates produced clear plaques with diameters ranging from 2.0 ± 0.3 to 8.0 ± 0.8 mm against *S*. Pullorum strain SP01. Lytic activity of the phages against various bacterial strains was analyzed to screen phages with broad host range. The phages showed lytic activity against three to nine different serovars of *Salmonella*. Among them, phage SHWT1 had the broadest host range, with the capacity to lyse nine serovars of *Salmonella* at different lytic rates. However, no lytic activity was observed against the other species tested in this study, including *Escherichia coli* and *Staphylococcus aureus* (Table 1). When infecting the prevalent *Salmonella* serovars *S*. Pullorum, *S*. Gallinarum, *S*. Enteritidis, *S*. Typhimurium, phage SHWT1 formed clear plaques with diameters of 3.0 ± 0.4 to 8.0 ± 0.8 mm (Figure 1A). Phage SHWT1 was therefore selected for further analysis.

**Table 1:** The host range of phage SHWT1.

**Bacteria and serovars**	**No. of strains**	**No. of Lytic strains**
*S*. Pullorum	53	46
*S*. Gallinarum	6	5
*S*. Enteritidis	7	4
*S*. Typhimurium	14	12
*S*. Derby	4	1
*S*. London	1	1
*S*. Agona	1	0
*S*. Typhi	2	1
*S*. Dublin	1	0
*S*. Heidelberg	1	1
*S*. Paratyphi A	1	0
*S*. Paratyphi B	1	1
*S*. Paratyphi C	1	0
*Escherichia coli*	10	0
*Staphylococcus aureus*	5	0

### Morphology of Phage SHWT1

TEM analysis of purified SHWT1 particles revealed a structure comprising a polyhedral head with diameter 52.8 ± 2.3 nm and a non-contractile tail of 122.0 ± 2.8 nm (Figure 1B). Based on morphological features, phage SHWT1 belongs to the family *Siphoviridae* in the order *Caudovirales* according to the guidelines of the International Committee on Taxonomy of Viruses (50).

### Determination of Optimal MOI of Phage SHWT1

Host *Salmonella* strains were infected with phage SHWT1 at various ratios, and the phage titer was tested to determine the optimal MOI. Based on the maximum phage titer obtained, the optimal MOIs of phage SHWT1 to *S*. Pullorum, *S*. Gallinarum, *S*. Enteritidis and *S*. Typhimurium, and were 0.001, 0.01, 100, and 0.1, respectively (Table 2).

**Table 2:** Optimal multiplicity of infection (MOI) of phage SHWT1.

***Salmonella* strains**	**Bacterial concentration (CFU/mL)**	**Phage titer (PFU/mL)**	**Optimal MOI**	**Phage titer after incubation (PFU/mL)**
*S*. Pullorum SP01	1 ×10^8^	1 ×10^5^	0.001	2 ×10^14^
*S*. Gullinarum SG02	1 ×10^8^	1 ×10^6^	0.01	1.08 ×10^10^
*S*. Enteritidis SE12	1 ×10^7^	1 ×10^9^	100	1.38 ×10^10^
*S*. Typhimurium SAT52	1 ×10^8^	1 ×10^7^	0.1	3 ×10^10^

### One-Step Growth Curve

**Figure 2:**
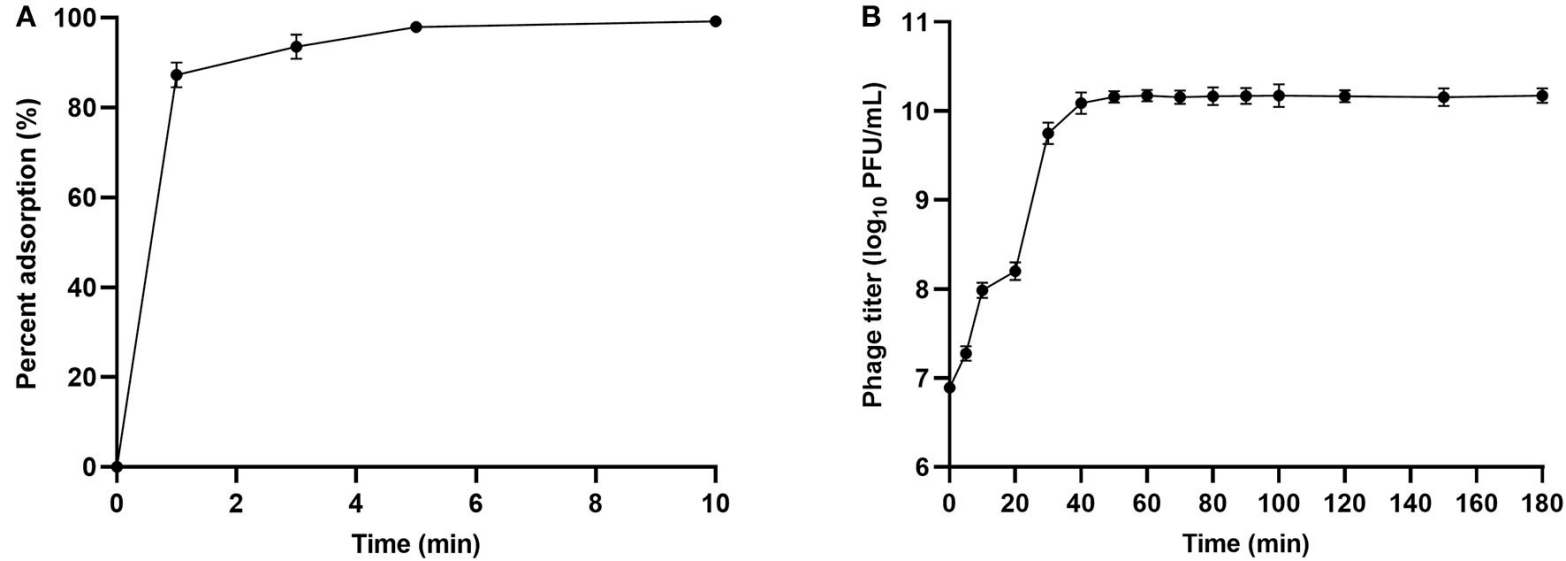
Adsorption rate and one-step growth curve of phage SHWT1. **(A)** Adsorption rate. Adsorption of phage SHWT1 to *S*. Pullorum SP01 was expressed as a percentage of the total phages added. **(B)** One-step growth curve of phage SHWT1 on *S*. Pullorum SP01.

We examined the adsorption ability of phage SHWT1 with host *S*. Pullorum SP01. The results showed that phage SHWT1 had an adsorption rate of 87.2% within 1 min, 93.6% within 3 min, 99% within 5 and 10 min, indicating that saturation of adsorption was reached to 99% after 5 min (Figure 2A). This result indicated the high and rapidly adsorption rate of phage SHWT1. The adsorption rate constant (*k* value) of SHWT1 to host *S*. Pullorum SP01 was 8.8 ± 0.5 ×10^−9^ mL/min for the 5 min time point.

The one-step growth curve produced for phage SHWT1 on *S*. Pullorum SP01 showed a latent period of ~5 min and a burst period of 35 min, with an average burst size of 146.6 ± 10.8 PFUs/cell. The titer of phage SHWT1 reached stationary phase after 50 min, with a titer of 10^10^ PFU/mL (Figure 2B).

### Thermal Stability and pH Sensitivity of Phage SHWT1

**Figure 3:**
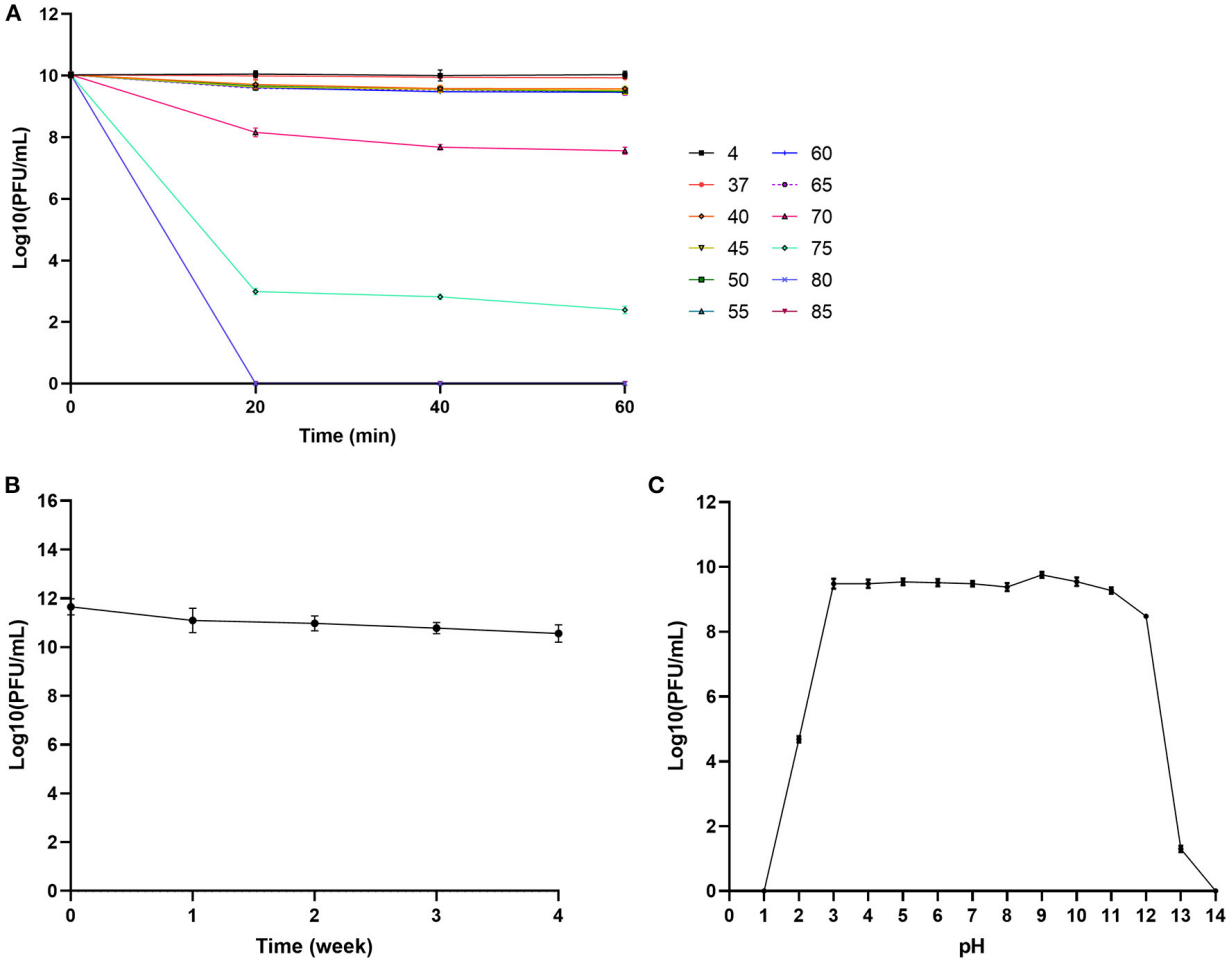
Stability of phage SHWT1 under different temperature and pH. **(A)** Thermal stability. Phage SHWT1 was stable from 4 to 65°C, but its stability decreased above 70°C. **(B)** Room temperature storage test. Phage SHWT1 maintained its activity for 4 weeks when stored at room temperature. **(C)** pH stability. Phage SHWT1 exhibited pH stability range from 3 to 12. Values are means of three repeats with standard deviations.

Thermal stability tests indicated that phage SHWT1 survived incubation for 1 h at 4 to 65°C. The phage titer continuously decreased when the incubation temperature was above 70°C. Moreover, the phage was inactivated within 20 min in 80°C thermal condition (Figure 3A). Phage SHWT1 maintained activity over 4 weeks when stored at room temperature (Figure 3B), demonstrating its ability to tolerate normal temperature environments and its suitability for potential future applications.

pH sensitivity tests showed that SHWT1 maintained activity at pH 3 to pH 12, but the titers dramatically decreased at pH 2 and pH 13. No surviving phages were observed at pH 1 or pH 14 (Figure 3C).

### Inhibition of *Salmonella* Planktonic Cells by Phage SHWT1 *in vitro*

**Figure 4:**
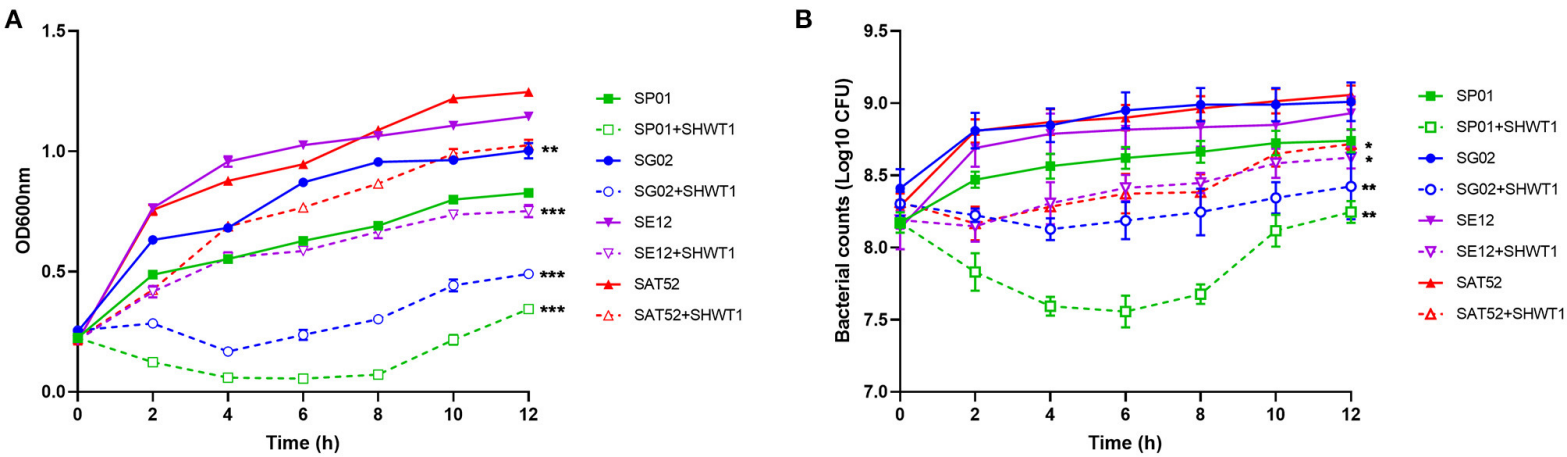
Inhibition of *Salmonella* planktonic cells by phage SHWT1 *in vitro*. Each *Salmonella* were incubated with or without phage SHWT1. The growth **(A)** and bacterial counts **(B)** of the *Salmonella* were significantly inhibited by phage SHWT1. Statistical significance was assessed using two-way ANOVA (*P < 0.05; **P < 0.01; ***P < 0.001).

To evaluate the antibacterial effect of phage SHWT1 *in vitro*, the different serovars of *Salmonella* were treated with SHWT1 at optimal MOIs. The bacterial planktonic cells growth was efficiently inhibited by SHWT1 (*P* < 0.01 and *P* < 0.001) (Figure 4A). Moreover, phage SHWT1 treatment lead to significant reductions in the number of living bacterial cells of each *Salmonella* serovars (*P* < 0.05 and *P* < 0.01) (Figure 4B). These experiments generally corroborated the conclusions that phage SHWT1 was able to lyse the *Salmonella* planktonic cells.

### Control of Biofilm Formation by Phage SHWT1

**Figure 5:**
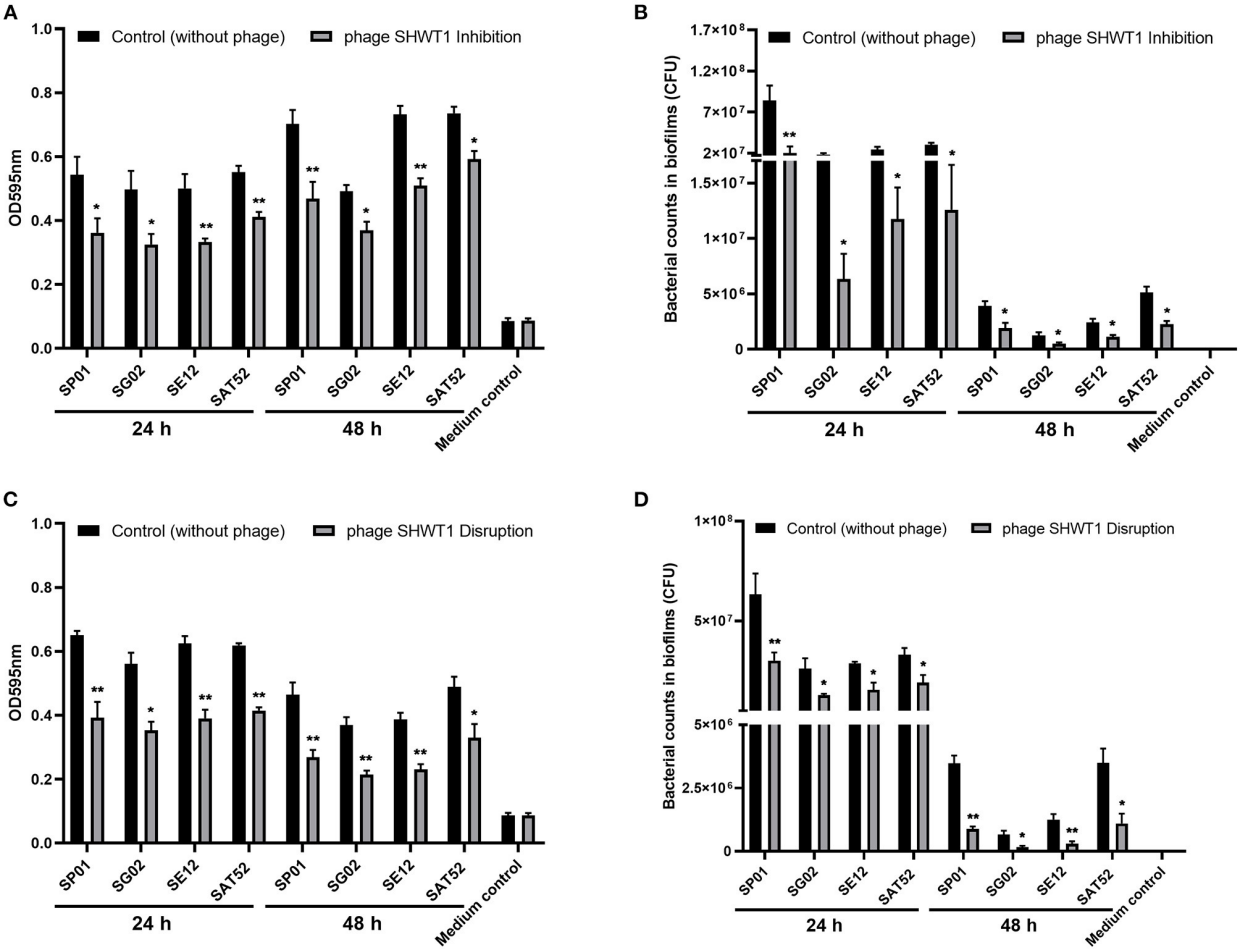
The effect of phage SHWT1 on biofilm formation by *Salmonella* was tested as described in “Materials and Methods.” The biofilm formation **(A)** and bacterial counts within biofilm **(B)** were significant inhibited when treating with phage SHWT1. Moreover, the phage SHWT1 could significant eliminate the formed biofilm **(C)** and lyse the bacterial cells **(D)**. Statistical significance was assessed using one-way ANOVA (*P < 0.05; **P < 0.01).

Given that most bacteria in clinical infections reside in biofilms, the activity of phage SHWT1 in inhibiting biofilm formation was determined by crystal violet staining in 96-well plates. Biofilm formation by *S*. Pullorum, *S*. Gallinarum, *S*. Enteritidis, and *S*. Typhimurium was significantly inhibited when treating with phage SHWT1 compared with the negative control (*P* < 0.05 and *P* < 0.01) (Figure 5A). The bacterial numbers within the biofilms were estimated in a similar experiment. Consistently, treatment of phage SHWT1 led to the significant reductions in viable counts of *Salmonella* within biofilm (*P* < 0.05 and *P* < 0.01) (Figure 5B). In addition, the capacity to disrupt biofilms of phage SHWT1 was tested. We found that phage SHWT1 was able to eliminate the formed biofilm and lyse the bacterial cells (*P* < 0.05 and *P* < 0.01) (Figures 5C,D).

### Intracellular Lytic Activity of Phage SHWT1

**Figure 6:**
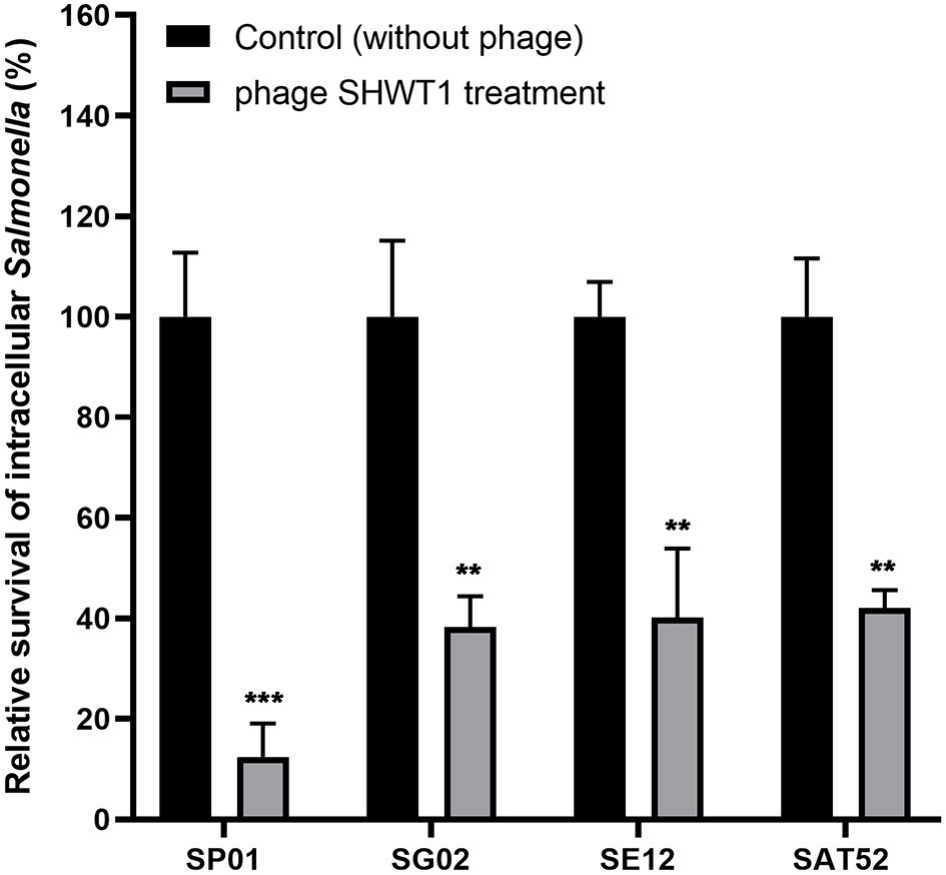
Phage SHWT1 eliminates intracellular *Salmonella* within macrophages. Chicken macrophage HD11 cells were infected with each *Salmonella*, and the cells were incubated with phage SHWT1 for 12 h to kill the intracellular *Salmonella*. The relative survival of intracellular *Salmonella* in HD11 cells was expressed compared with the untreatment group. Statistical significance was assessed using one-way ANOVA (**P < 0.01; ***P < 0.001).

The lytic activity of phage SHWT1 against the intracellular *Salmonella* was evaluated, and result in terms of percent survival was shown in Figure 6. After 12 h of phage treatment, *S*. Pullorum, *S*. Gallinarum, *S*. Enteritidis, and *S*. Typhimurium showed 87.6, 61.7, 57.9, and 59.9% reductions in intracellular survival, respectively (*P* < 0.01 and *P* < 0.001). This observation indicated that phage SHWT1 was able to lyse intracellular *Salmonella* of macrophages.

### Genomic and Phylogenetic Analysis

**Figure 7:**
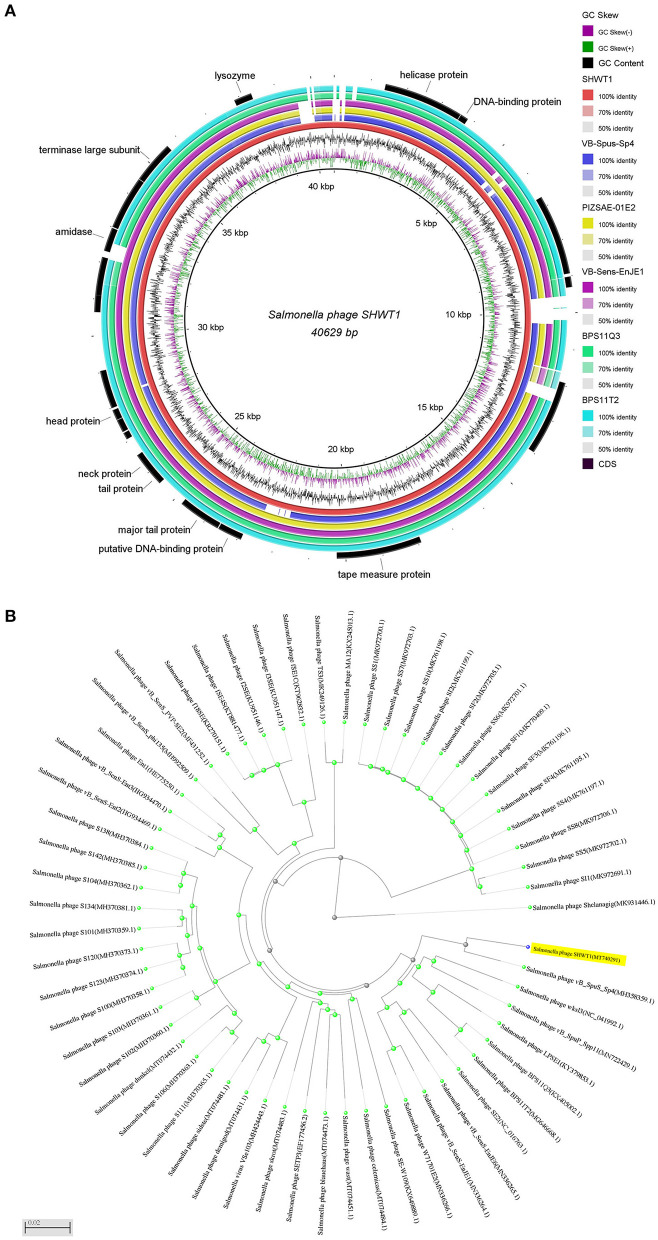
Genomic and phylogenetic analysis of phage SHWT1. **(A)** Genome map of phage SHWT1. The genomic organization of phage SHWT1 was compared to *Salmonella* phages vB-SpuS-Sp4, PIZSAE-01E2, vB-SenS-EnJE1, BPS11Q3, and BPS11T2. Genome arrangements were divided into several circles. The two inner circles represent the GC skew of G-C/G+C as purple and green and GC content, respectively. The full length genome of phage SHWT1 were showed as red circle. The coincident regions of other *Salmonella* phages were displayed, and the blank was the non-coincident region. The outermost circle represents the important functional proteins of phage SHWT1. **(B)** Phylogenetic analysis of *Salmonella* phages. Phylogenetic trees were constructed based on whole genome sequences of *Salmonella* phages by NCBI BLASTN algorithm. GenBank accession numbers are also provided. Phage SHWT1 is closely related to phage vB-SpuS-Sp4.

The genome of phage SHWT1 comprises 40,629 bp of double-stranded DNA, with a GC content of 49.83%. Putative ORFs were predicted using GeneMarkS, and 56 ORFs were identified (Supplementary Table 2), with 18 ORFs present on the positive strand and 38 ORFs on the negative strand. Among the 56 ORFs, only 21 (37.5%) had annotated functions, while 35 ORFs (62.5%) were assigned as hypothetical proteins. The identified ORFs were categorized into four functional groups, including DNA replication/modification (primase/helicase, DNA binding protein, DNA polymerase, DNA cytosine methyltransferase), structural components (tail spike protein, tape measure protein, tail protein, neck protein, head-tail joining protein, head protein, coat protein, head morphogenesis protein), packaging module (portal protein, terminase large subunit), and host lysis (lysozyme) (Figure 7A). There was no tRNA gene in phage SHWT1, suggesting that this phage is completely dependent on the host tRNA for protein synthesis. Toxin genes, virulence genes, and antibiotic resistance genes were not detected in the genome of phage SHWT1 through the genomic sequence analysis using online database. In addition, no putative integrase genes were identified, indicating that phage SHWT1 could not integrate into the host bacterial genome and might be lytic in nature (39). Thus, phage SHWT1 is potentially a safe agent for controlling *Salmonella* infection.

Whole genome sequence analysis revealed that phage SHWT1 is a member of the subfamily *Guernseyvirinae*, genus *Jerseyvirus*. The genome of phage SHWT1 was homologous to those of other *Salmonella* phages. Phage SHWT1 was most closely related to phage vB-SpuS-Sp4 (GenBank accession no. MH358359.1), with a 97% sequence similarity based on 90% query coverage, which formed a distinct branch within the clade (Figure 7B). The genome of phage vB-SpuS-Sp4 has been sequenced and is a 43,614 bp circular DNA, with 67 predicted ORFs. However, the characteristics of phage vB-SpuS-Sp4 are unknown. Other phages showed an average sequence similarity below 93% with SHWT1.

### The Therapeutic Effect of Phage SHWT1

**Figure 8:**
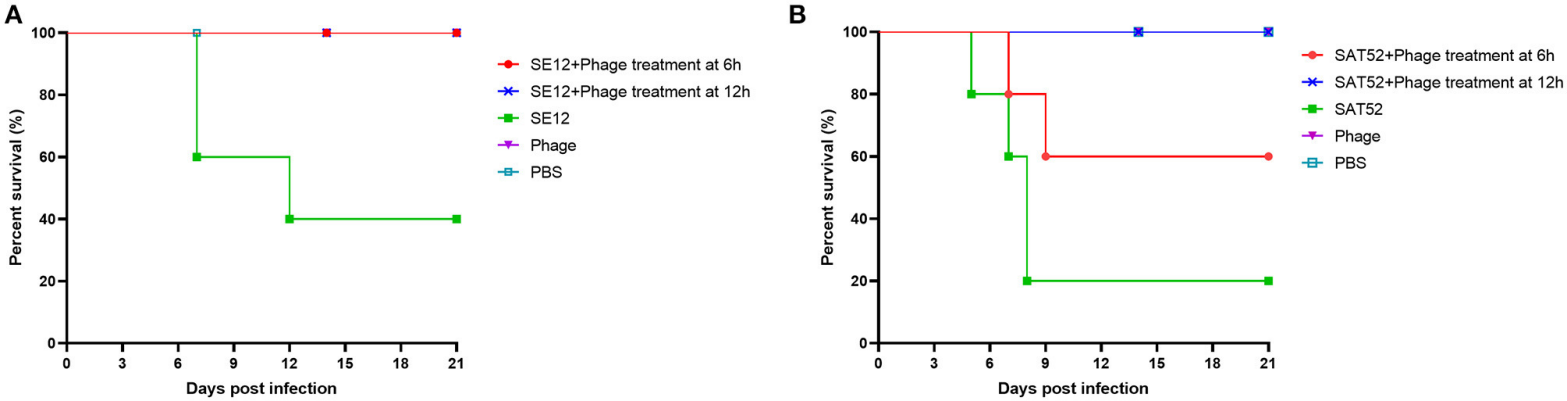
The therapeutic effect of phage SHWT1 against *Salmonella* infection. Mice were orally infected with *S*. Enteritidis SE12 **(A)** or *S*. Typhimurium SAT52 **(B)**. Then, mice were treatment with phage SHWT1 at 6 or 12 h post-infection. Mice injected with *Salmonella*, phage or PBS only was used as control groups. The survival rates of mice were monitored. The phage SHWT1 successfully cures *S*. Enteritidis and *S*. Typhimurium infections in mice.

The therapeutic efficacy of phage SHWT1 against the *S*. Enteritidis and *S*. Typhimurium infection in a mouse model was tested. The results showed that reduced mortality was observed when phage treatment was introduced. The survival rate of S. Enteritidis infection group is 40%, however, all the mice survival by the phage SHWT1 therapy. Moreover, phage SHWT1 could rescue 40 and 80% mice from *S*. Typhimurium infection when treatment at 6 or 12 h post-infection. The health and survival of phage control group was same to the PBS control group (Figure 8), indicating the safety of phage SHWT1.

## Discussion

With the development and spread of antibiotic-resistant bacteria, it is imperative to explore novel or alternative therapeutics against bacterial infections. The worldwide abundance, safety, high specificity, and environmentally friendly characteristics of phages render them ideal candidates to combat drug-resistant bacterial infections. The concept of phage therapy for controlling bacterial infections has been widely accepted (16–18). However, although numerous studies have described the applications of phages to inhibit bacterial infections, the relatively narrow lytic spectrum of phages is an important challenge to their further application (30, 31). Consequently, it is necessary to isolate novel and sensitive phages and determine their physiological and genomic characteristics to enrich the phage arsenal.

In this study, 68 lytic phages infecting *Salmonella* were isolated from wastewater of poultry farms in eastern China. Bacteriophages belonging to different families, including *Myoviridae, Siphoviridae, Microviridae*, and *Podoviridae*, are widely distributed in wastewater due to the presence of a vast array of host microorganisms (42, 51). Among the 68 lytic phages, a broad-host-spectrum phage, SHWT1, against various serovars of *Salmonella* was identified and characterized. Morphological and genome analysis showed that phage SHWT1 was a member of the family *Siphoviridae*, subfamily *Guernseyvirinae*, genus *Jerseyvirus*. Biological characteristics such as the latent period, burst size of phage, and stability in different environments are key factors for therapeutic applications of phages. A short latent period and high burst size were reported to facilitate effective killing of bacteria (52). Phage SHWT1 had a short latent period (5 min) and an average burst size of 146.6 ± 10.8 PFUs/cell, suggesting its potential as a bacterial treatment. Environmental pH and temperature affect phage activity and stability, thus it is essential that these parameters are tested to determine environment setups for phage application. Phage SHWT1 retained lytic activity for at least 60 min at temperatures ranging between 4 and 65°C. The stability of phage SHWT1 at room temperature for a month, suggested its suitability for practical applications. *Salmonella* infections commonly occur in the intestine where the pH is acidic. The efficacy of phage therapy via oral administration might be poor if the phage is unable to survive in the low gastric pH environment. Thus, it is necessary to determine the pH stability of the phage. Phage SHWT1 was stable at pH 3 to 12. These temperature and pH assays demonstrated that phage SHWT1 was tolerant to heat and extreme pH conditions, and these are appealing and unique characteristics for potential applications of the phage.

As a natural biological antagonist of bacteria, having a broad host range is one of the most important criteria in phage selection and application (53). Phage SHWT1 could lyse nine serovars of *Salmonella* including the prevalent serovars *S*. Pullorum, *S*. Gallinarum, *S*. Enteritidis, and *S*. Typhimurium. No lytic activity was observed against the tested bacterial strains of normal intestinal flora. Different host ranges have been reported for *Salmonella* phages of the family *Siphoviridae* (35, 36). A previous study indicated that *Siphoviridae* phages were considered as restricted-host-range bacteriophages (54). In contrast, it has been reported that some *Siphoviridae* phages simultaneously infect and lyse various strains of one species or many related bacteria (55, 56). The bacterial reduction test in this study indicated that growth of multidrug-resistant *Salmonella* was constantly inhibited for at least 8 h in the presence of phage SHWT1 at optimal MOIs. However, most antibiotics, including Erythromycin, Roxithromycin, Clindamycin, Cephradine, Spectinomycin, Doxycycline at 2 × minimum inhibitory concentration could not significantly reduce the viable cell density of multidrug-resistant *S*. Pullorum, *S*. Gallinarum, *S*. Enteritidis, and *S*. Typhimurium (data not shown). In addition, the ability to kill *Salmonella* within macrophages suggests the potential of phage SHWT1 therapies. Ideally, the therapeutic evaluation *in vivo* model is also needed for candidates for phage therapy. Previous studies have shown the bacterial infection can be cured by treatment with phage in experimentally or naturally infected animals (35, 57). Thus, we carried out the evaluation of the therapeutic efficacy of SHWT1 *in vivo* infection test. Although the efficacy of phage therapy by oral administration was poor due to the inactivation of phage in the stomach extreme pH conditions, our phage SHWT1 successfully protected mice against *S*. Enteritidis and *S*. Typhimurium infection. This is attribute to the acid tolerance characteristics of phage SHWT1. These results indicated that the effective therapeutic effect of phage SHWT1 for the multidrug-resistant *Salmonella* infection.

Biofilms provide a favorable environment for pathogens and contribute to multidrug resistance and persistent bacterial infections. Thus, biofilm formation is a significant problem as it renders bacterial infections more difficult to control (13, 14). Phages damage biofilms through various mechanisms, and these have been applied as biotechnologies in the treatment of infections and on the surfaces of medical devices (13, 58–60). Phage SHWT1 could inhibit biofilm formation and bacterial counts with biofilm of *Salmonella*. Furthermore, we found that phage SHWT1 was able to disrupt the *Salmonella* biofilms. However, the mechanism of action remains to be elucidated. This phage might directly degrade biofilms by killing bacteria during colonization onto the surface or it may act via an as yet unknown degradation mechanism. Previous studies similarly indicated that phages of the family *Podoviridae* not only inhibited bacterial growth but also decreased biofilm formation (36, 61, 62).

Phages acquire and contribute genes to other phage genomes as well as to bacterial genomes, and such genes are powerful factors in the evolution, physiology, and virulence of the host bacteria (31). Complete genome sequencing, annotation, and alignment demonstrated that phage SHWT1 possessed crusted functional regions, including domains for DNA replication/modification, structural components, packaging module, and host lysis; this was similar to other *Salmonella* phages (63). Since the geographical locations of these phages are different, the high homology might arise from the complex evolutionary relationships with their common host *Salmonella*. The genome of phage SHWT1 showed highest similarity to that of phage vB-SpuS-Sp4, which characteristics was unknown. The only characterized related phage wksl3 exhibited similar host range compared with phage SHWT1 (64), which might due to the homology of tail spike protein. However, there were some genomic rearrangements and distinct gene regions between these two phages. This indicated that SHWT1 was a novel *Salmonella* phage. Annotation of the genome of phage SHWT1 identified some important proteins for antibacterial activity, including lysozyme, which are essential proteins involved in host cell lysis (65, 66). Spread of antibiotic-resistant genes virulence genes via mobile genetic elements, such as plasmid, transposon, phage, contributes to the increase in multidrug-resistance and virulence of bacterial pathogen. Evidences had suggested that phage probably be a potential reservoir for the antibiotic-resistant genes, virulence genes acquisition and dissemination (31). Thus, characterization of phage genome sequences could be helpful in excluding the phages carrying harmful genes from phage therapy. In this study, toxin genes, virulence genes, antibiotic resistance genes, and integrase genes were not observed in the genome of phage SHWT1, demonstrating the potential safety of this phage if used as a therapeutic agent against *Salmonella* infection.

## Conclusion

In summary, a lytic phage, SHWT1, against multidrug-resistant *Salmonella* was isolated and characterized. Phage SHWT1 tolerates a range of pH and temperatures, and is able to effectively inhibit the growth, biofilm formation, bacterial counts within biofilm of the prevalent *Salmonella* serovars. Moreover, phage SHWT1 exhibits lytic activity against the intracellular *Salmonella* within macrophages. Genome analysis of phage SHWT1, herein, provides fundamental research for functional studies and supports the application of this phage in biocontrol. Especially, phage SHWT1 could successfully protect mice against *S*. Enteritidis and *S*. Typhimurium infection. With the prohibition of the use of antibiotics in poultry farm, phage SHWT1 has potential as an alternative therapeutic agent against salmonellosis caused by multidrug-resistant *Salmonella*.

## Data Availability Statement

The complete genome sequence of phage SHWT1 has been deposited in the GenBank database under accession number MT740291.1.

## Ethics Statement

The animal experiments were conducted in strict accordance with the guidelines of the Humane Treatment of Laboratory Animals and were approved by the Animal Care and Use Committee at the Shanghai Veterinary Research Institute (permit No: SHVRI- SV-20201014-04).

## Author Contributions

SW conceived the project. ZY isolated phages. CT, YZ, YW, HZ, and DA performed experiments to characterize phage SHWT1. CT wrote the original manuscript draft. TL, MT, JQ, CD, and SG analyzed and discussed the experimental results. SW and SY directed the experiments, funded the research, and edited the manuscript. All authors contributed to the article and approved the submitted version.

## Conflict of Interest

The authors declare that the research was conducted in the absence of any commercial or financial relationships that could be construed as a potential conflict of interest.
